# Low-dose dexmedetomidine improves postoperative sleep and pain in gynecological surgery: a randomized trial

**DOI:** 10.3389/fphar.2026.1766782

**Published:** 2026-03-05

**Authors:** Yi Zeng, Qing-Li Li, Rui Hu, Lei Chen, Yun-Wang Zhang, Sha Li, Fa-Bin Yang, Feng Liu, Jian-Hong Wu, Guo-Yi Gao, Ye-Tian Yang, Chao-Hui Zou

**Affiliations:** 1 Department of Anesthesiology, The People’s Hospital of Xishuangbanna Dai Nationality Autonomous Prefecture, Jinghong, Yunnan, China; 2 Department of Gynaecology, The People’s Hospital of Xishuangbanna Dai Nationality Autonomous Prefecture, Jinghong, Yunnan, China; 3 Traditional Chinese Medicine and Proctology, The People’s Hospital of Xishuangbanna Dai Nationality Autonomous Prefecture, Jinghong, Yunnan, China; 4 Department of Anesthesiology, The Affliated Yan’an Hospital of Kunming Medical University, Kunming, Yunnan, China

**Keywords:** dexmedetomidine, gynecologic surgery, multimodal analgesia, postoperative pain, postoperative sleep disorders, sufentanil

## Abstract

**Background:**

Postoperative sleep disturbances often lead to a vicious cycle with pain, severely hindering the recovery of patients. Women, due to fluctuations in sex hormones and their unique pain modulation mechanisms, are particularly vulnerable to both postoperative sleep disorders and pain. Dexmedetomidine (DEX) has shown potential in promoting sleep and providing analgesia. Therefore, exploring its application in optimizing postoperative pain management for gynecological patients is of great significance in enhancing recovery outcomes.

**Objective:**

This study aimed to assess the impact of adding low-dose dexmedetomidine (DEX) to a sufentanil-based patient-controlled intravenous analgesia (PCIA) regimen on postoperative sleep quality and pain in patients undergoing gynecological surgery.

**Methods:**

This single-center, randomized, double-blind, placebo-controlled trial was conducted between 28 September 2025, and 30 November 2025. A total of 130 patients scheduled for elective gynecological surgery were enrolled. Participants were randomly assigned to one of two groups (65 patients per group) using a computer-generated randomization sequence, with allocation concealed via sequentially numbered, opaque sealed envelopes. Patients in the experimental (DS) group received a PCIA) regimen consisting of DEX (0.06 μg/kg/h) combined with sufentanil (0.04 μg/kg/h). The control (S) group received PCIA with sufentanil alone at the same dosage of 0.04 μg/kg/h.The primary outcome was the incidence of sleep disturbance on the first postoperative night, defined as a Pittsburgh Sleep Quality Index (PSQI) global score ≥5. Secondary outcomes included PSQI scores on the first and second postoperative nights, Visual Analogue Scale (VAS) pain scores assessed at 6, 12, 24, and 48 h postoperatively, total postoperative sufentanil consumption, the number of PCA button presses recorded by the infusion pump, and the incidence of adverse events—specifically bradycardia (heart rate <50 bpm), hypotension (mean arterial pressure <60 mmHg), postoperative nausea and vomiting (PONV), and the use of rescue analgesia with intravenous flurbiprofen axetil.

**Results:**

For the primary outcome, the incidence of sleep disturbance (PSQI ≥ 5) was significantly lower in the DS group compared to the S group (21.5% vs. 47.7%, P = 0.002). Regarding secondary outcomes, PSQI scores on the first and second postoperative nights were also significantly better in the DS group (P = 0.020 and P = 0.016, respectively). In terms of pain control, VAS pain scores at all time points within 48 h were significantly lower in the DS group (P < 0.05). However, there were no significant differences between the two groups in sufentanil consumption (P = 0.593) or the number of PCA presses (P = 0.092) during the 48-h postoperative period. For adverse events, the DS group had a significantly higher incidence of bradycardia (16.9% vs. 3.1%, P = 0.009), a significantly lower incidence of postoperative nausea and vomiting (PONV) (13.8% vs. 32.3%, P = 0.013), and a lower proportion of patients requiring rescue analgesia (9.2% vs. 26.2%, P = 0.011). No significant difference was observed in the incidence of hypotension between the groups (6.2% vs. 4.6%, P = 0.676).

**Conclusion:**

The addition of low-dose DEX (0.06 μg/kg/h) to a sufentanil-based PCIA regimen significantly improved postoperative sleep quality, reduced pain, and decreased the incidence of PONV in gynecological patients, without reducing opioid consumption. The mechanism may be attributed to DEX’s mood-stabilizing and direct sleep-promoting effects rather than an opioid-sparing effect. Although the risk of bradycardia increased, there was no rise in hypotension risk. Collectively, our findings support that this low-dose regimen is a safe and effective multimodal analgesic strategy, offering a valuable therapeutic option to simultaneously improve sleep and alleviate pain in female patients during the postoperative period.

**Clinical Trial Registration:**

https://www.chictr.org.cn, identifier ChiCTR2500108204.

## 1 Introduction

Sleep problems have become a global public health concern with increased cardiovascular, metabolic and cognitive impairment risk (Grandner, 2022; Li et al., 2022; Olivares et al., 2022). Postoperative patients are at risk for entering a vicious cycle whereby pain disrupts sleep and poor sleep heightens one’s sensitivity to pain. This key bidirectional relationship is critical because just one night of sleep deprivation is enough to hinder endogenous pain inhibition and induce hyperalgesia (Miller et al., 2015).

Women seem disproportionately impacted. Sleep disorders occur significantly more in females than males as often demonstrated in epidemiological studies (Chattu et al., 2018). Furthermore, experimental data show that the effect of sleep loss on pain threshold is greater in women than in men (Eichhorn et al., 2018). This greater vulnerability probably arises from several sex-specific factors, including cyclical changes in sex hormone levels (e.g., estrogen, progesterone) throughout the lifespan (Pengo et al., 2018; Tobias et al., 2021), and sex-specific neuroimmune mechanisms of pain modulation (Sorge et al., 2015). In addition, sexual dimorphism exists in pain relief drug action which may impact postoperative opioid doses (Cepeda and Carr, 2003; Xu Q. et al., 2024).

The per-operative environment itself can cause sleep disruption (Adams et al., 2024). The sleep homeostasis is disturbed when the γ-aminobutyric acid (GABA) and N-methyl-D-aspartate (NMDA) receptors which are centrally acting and located in the central nervous system (CNS) are affected with the help of anesthesia in the body resulting in non-physiological activity in the brain (Moody et al., 2021; Yang et al., 2022). Preclinical results also demonstrate that dexmedetomidine and other common anaesthetics affect circadian clock genes expressed in brain, and increase fragmentation of sleep-wake cycles in the postoperative period (Mizuno et al., 2022).

Dexmedetomidine (DEX) is a selective α2-adrenoceptor agonist that offers interesting properties for addressing this complex problem. It induces analgesia and anxiolysis and uniquely promotes sleep architecture often enhanced by many commonly used anaesthetics, particularly stages of non-rapid eye movement (NREM) sleep (Weerink et al., 2017; Akeju et al., 2018). One systematic review reports more DEX during and after surgery can improve sleep quality and reduce pain (Huang et al., 2021). Women have been reported to be at a higher risk for sleep pain comorbidity after surgery. It has also been claimed that sex hormones can modulate pain pathways and opioid efficacy (Fillingim and Gear, 2004). The specific role of DEX as adjunct to opioid analgesia in gynecological patients needs to be evaluated. Although the Pittsburgh Sleep Quality Index (PSQI) was primarily designed for assessing chronic sleep quality, it has recently been applied in the evaluation of early postoperative sleep, including the first and second nights following surgery. For instance, PSQI has been utilized in maxillofacial and gastrointestinal surgical settings to capture short-term sleep changes analogous to our study design (Wang et al., 2023; Yan et al., 2023). This randomized controlled trial was conducted to analyze the effect of low-dose DEX as an additive to a sufentanil-based patient-controlled intravenous analgesia (PCIA) on postoperative sleep quality along with pain in patients undergoing gynecological surgery.

## 2 Materials and methods

### 2.1 Study design

This single-center, randomized, controlled, double-blind trial adhered to the ethical principles of the Declaration of Helsinki and local regulations. Prior to initiation, the study protocol was approved by the Medical Ethics Committee of Xishuangbanna Dai Autonomous Prefecture People’s Hospital (Approval No. 2025022; Date: 27 March 2025) and registered with the Chinese Clinical Trial Registry (https://www.chictr.org.cn; Registration No. ChiCTR2500108204; Date: 26 August 2025). Written informed consent was obtained from all participants before enrollment. The study’s implementation and reporting followed the CONSORT guidelines to ensure scientific rigor and transparency.

Female patients scheduled for elective gynecological surgery between 28 September 2025 and 30 November 2025 were enrolled. Eligible procedures included both laparoscopic and open surgeries for uterine, fallopian tube, or ovarian pathologies. The inclusion criteria were as follows: (1) American Society of Anesthesiologists (ASA) physical status classification I-II; (2) body mass index (BMI) between 18.5 kg/m^2^ and 30 kg/m^2^; (3) female patients aged 18–65 years; (4) voluntary postoperative use of intravenous patient-controlled analgesia (PCIA). The exclusion criteria were: (1) long-term preoperative use of sedatives/analgesics or a history of chronic pain; (2) severe cardiopulmonary disease (e.g., sick sinus syndrome, atrioventricular block, severe sinus bradycardia with heart rate <50 beats per minute, uncontrolled hypertension, obstructive sleep apnea-hypopnea syndrome, chronic obstructive pulmonary disease); (3) severe hepatic or renal dysfunction; (4) known allergy to DEX or sufentanil; (5) preoperative Pittsburgh Sleep Quality Index (PSQI) score ≥5; (6) pregnancy or lactation; (7) intraoperative blood loss ≥500 mL or surgical duration ≥2 h.

### 2.2 Randomization and blinding

A double-blind design was implemented using a sealed envelope method. An independent researcher generated a computer-based random sequence, and the group allocation results (experimental/control) were sealed in sequentially numbered, opaque envelopes. A post-anesthesia care unit (PACU) nurse, who was not involved in postoperative follow-up, opened the corresponding numbered envelope and prepared the analgesic pump according to the allocated group and the patient’s weight. The labels on the pumps containing analgesia were only the study identification numbers, thus anesthesiologists and patients and data collectors were blinded to group assignment. An experienced nurse, blind to group allocation, assessed the Pittsburgh Sleep Quality Index (PSQI) scores pre-intervention and the Visual Analogue Scale (VAS) pain scores post-intervention. Adverse events were recorded during effective blinding. Nurses documented the data according to the pain pump identification number. Only the principal investigator had an unblinding envelope, which was to be opened in case of a severe adverse event. To manage pain, the patients in the experimental group (DS group) received a PCIA which had DEX (3 μg/kg), and sufentanil (2 μg/kg) and ondansetron (8 mg) while the patients in the S group received sufentanil (2 μg/kg) and ondansetron (8 mg) similar to the DS group. It was then diluted in normal saline so that the total volume was 100 mL. The two solutions in volume appearance and infusion parameters were identical. Background infusion was set at 2 mL/h with a bolus dose of 0.5 mL and 15-min lockout period. To promote blinding, both solutions were matched in every possible way. During the preoperative visit, we teach all patients how to assess their postoperative pain and operate the analgesia pump, including what button to push to use the bolus.

### 2.3 Anesthesia and analgesia

Every patient was anesthetized using a standardized balanced general anaesthesia technique, intravenous and inhalational. Patients were asked to stop eating 8 hours before the surgery but can drink clear liquids up to 2 h before. To maintain hemodynamic stability, 20 mL/kg bolus of lactated Ringer’s solution was given prior to induction of anesthesia. Intravenous propofol (1.5–2.5 mg/kg), cisatracurium (0.2 mg/kg), and sufentanil (3 μg/kg) were used for anesthesia induction. The anesthetic maintenance involved a continuous infusion of propofol (4–6 mg/kg/h) and remifentanil (0.1–0.25 μg/kg/min), supplemented with inhalation of 2%–3% sevoflurane. We evaluated the depth of anesthesia by continuously monitoring Bispectral Index (BIS) with a target range of 40–55 and infusion rate modification in response to surgical stimulus. While surgery was going on blood pressure (BP) and heart rate (HR) were maintained at ±20% of baseline and end-tidal carbon dioxide (ETCO_2_) was maintained at 35–45 mmHg. Patients were given dexamethasone (8 mg) and ondansetron (4 mg) preoperatively to prevent postoperative nausea and vomiting (PONV). A supplementary dose of sufentanil (0.05 μg/kg) was administered 30 min before the expected surgical closure to avoid acute pain during recovery. All anesthesia agents were stopped at the end of the operation. Both anesthesia and surgical durations were recorded. Once extubation occurred and spontaneous ventilation was resumed, the patients were transferred to the post-anesthesia care unit (PACU) where PCA pumps pre-set according to the randomization protocol were started with only the background infusion. No loading dose was administered. After the criteria were met (Steward score was ≥5.), patients were transferred back to the surgical ward from the recovery ward.

### 2.4 Outcome measures

The incidence of sleep disturbance on the first postoperative night assessed by PSQI global score ≥5 was the primary outcome. We used the Pittsburgh Sleep Quality Index (PSQI) to assess subjective sleep quality (Buysse et al., 1989). The PSQI is a validated 19-item self-report questionnaire measuring seven components. Each component is scored from 0 to 3. The components evaluate subjective sleep quality, sleep latency, sleep duration, sleep efficiency, sleep disturbance, use of hypnotic medications, and daytime dysfunction. Subjective sleep quality refers to whether the subject feels their sleep is adequate. Sleep latency means the time taken to fall asleep after lying down. Sleep duration is the total amount of sleep the subject gets. Sleep efficiency is how much of the time spent in bed is spent asleep. Sleep disturbance means whether the subject wakes up during the night or has bizarre dreams. Use of hypnotic medications means whether the subject takes any medication to help them sleep. Daytime dysfunction refers to whether the subject sometimes feels fatigued and is unable to concentrate. The global scores range from 0 to 21, with a higher score representing poorer sleep quality. Patients completed the PSQI both preoperatively and on the first and second postoperative days. During the preoperative visit, detailed instructions were provided to them to complete this questionnaire.

### 2.5 Postoperative pain assessment

The Intensity of Pain was measured using Visual Analogue Scale (VAS) (Li et al., 2007), a simple technique using 10 cm line on which patient marks their pain intensity. It quantifies intensity of pain along 10 cm line (0: no pain and 10: worst imaginable pain). According to this scale, pain was categorized as 0 (no pain), 1–3 (mild pain), 4–6 (moderate pain), 7–10(severe pain). Session VAS was assessed for each patient at the intervals of post-operative 6, 12, 24, and 48 h under supervision of trained staff. If the VAS score was persistently ≥4 after two attempts at PCA bolus subsequent bolus delivery, rescue analgesia with intravenous flurbiprofen axetil (50 mg) was given following nurse assessment.

### 2.6 Secondary outcomes

Additional secondary outcomes were monitored and defined as follows:

1. Bradycardia was defined as a heart rate below 50 beats per minute. The condition must last for 5 min or more to be considered serious. The protocol for giving atropine, as laid down in the study, was a heart rate (HR) of less than 40 bpm, or an HR of less than 50 bpm with symptoms of hypoperfusion (e.g., lightheadedness, syncope) or hypotension (mean arterial pressure <60 mmHg).
2. Hypotension was defined as a MAP < 60 mmHg or a reduction of >20% from the baseline value lasting for 5 min. Intervention with ephedrine was required if MAP dropped below 55 mmHg or if hypotension was associated with signs of organ malperfusion.
3. If the patient complained about nausea, retching, or vomiting during the first 48 postoperative hours, then postoperative nausea and vomiting (PONV) was recorded. Regardless of whether antiemetic rescue medication was administered.
4. The administration of IV flurbiprofen axetil (50 mg) after assessment by the nurse, when the pain was prolonged and evaluated to be inadequate (persistent VAS ≥ 4 after two attempts at PCA bolus).

## 3 Sample size calculation

Sample size determination referenced Duan et al. (2020).'s cohort study evaluating DEX’s sleep-protective effects in non-cardiac surgery. Analysis of gynecological subgroups (n = 576) revealed postoperative sleep disturbance incidences of 31.4% in controls versus 8.6% in the low-dose DEX group (P < 0.001). With an absolute intergroup difference (Δ) of 22.8%, α = 0.05 (two-tailed), and 90% power, the two-independent-proportions formula indicated 63 patients per group. Accounting for an anticipated 10% dropout rate (due to postoperative loss-to-follow-up or data incompleteness), 70 patients per group (total N = 140) were enrolled.

## 4 Statistical analysis

Analyses utilized SPSS 25.0 (IBM Corp., Armonk, NY). Normality was assessed via Shapiro-Wilk testing. Continuous data are presented as mean ± SD. Non-normally distributed variables (postoperative PSQI scores, sufentanil consumption, PCA demand attempts) underwent rank analysis of covariance (Rank ANCOVA) with Rank-transformation to address non-normality, controlling for surgical type and baseline PSQI as covariates. Between-group PSQI comparisons at two postoperative timepoints employed Bonferroni-corrected independent t-tests following Levene’s test for homogeneity (α = 0.05). Nonparametric VAS scores were analyzed using linear mixed models incorporating group, surgery type, timepoint, and interaction terms as fixed effects, with subject-specific random intercepts to address repeated measures. Restricted maximum likelihood estimation and Satterthwaite degrees of freedom approximation ensured robustness against heteroscedasticity. Categorical variables were compared using χ^2^ or Fisher’s exact tests. Hierarchical Bonferroni correction controlled type I error for adverse events: tier-1 (serious events: bradycardia/hypotension) and tier-2 (general events: PONV/inadequate analgesia) used α = 0.025 (0.05/2). The primary outcome (Postoperative Nights 1 sleep disturbance) was tested at α = 0.05, with P < 0.05 considered significant.

## 5 Results

### 5.1 Patient demographics and clinical characteristics

**FIGURE 1:**
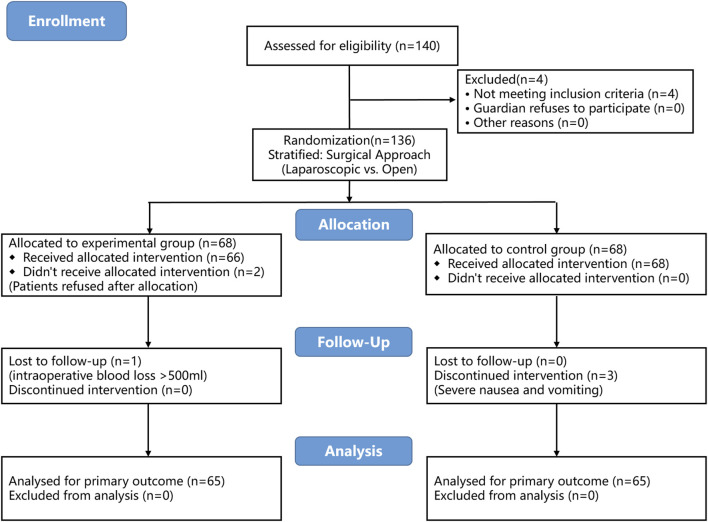
CONSORT flow diagram. CONSORT, Consolidated Standards of Reporting Trials.

Of the 140 patients initially enrolled, 4 were excluded during screening for not meeting the inclusion criteria. No guardians refused participation, and no exclusions occurred for other reasons. Following randomization, 2 patients in the DS group withdrew informed consent prior to anesthesia induction, and 1 patient in the same group was excluded due to intraoperative blood loss exceeding 500 mL. In the S group, 3 patients were withdrawn from the study because of severe postoperative nausea and vomiting during PCA use that persisted despite multiple medical interventions, necessitating discontinuation of postoperative analgesia. Consequently, 130 patients ultimately completed the study (Figure 1). The demographic and clinical characteristics such as age, BMI, ASA classification, surgical approach, operation time, anesthesia time, and intraoperative blood loss were balanced between the two groups. As expected with proper randomization, no statistical testing was performed on baseline variables (Table 1).

**TABLE 1:** Baseline characteristics and perioperative data of patients.

Characteristics	Experimental group (n = 65)	Control group (n = 65)
Age (years, mean ± SD)	46.6 ± 6.2	45.6 ± 5.8
Height (cm, mean ± SD)	156.3 ± 4.7	157.4 ± 3.1
Weight (kg, mean ± SD)	58.3 ± 7.6	58.1 ± 6.7
BMI (kg/m^2^, mean ± SD)	23.8 ± 3.1	23.3 ± 2.5
ASA grade, n (%)		
- Grade I	11 (16.9%)	14 (21.5%)
- Grade II	54 (83.1%)	51 (78.5%)
Surgical approach, n (%)		
- Laparoscopic surgery	48 (73.8%)	49 (75.4%)
- Open surgery	17 (26.2%)	16 (24.6%)
Operation time (min, mean ± SD)	84.8 ± 11.8	87.9 ± 13.7
Anesthesia time (min, mean ± SD)	102.1 ± 11.5	100.8 ± 12.2
Intraoperative blood loss (mL, mean ± SD)	118.5 ± 55.1	115.2 ± 50.5

Data are presented as mean ± SD, or n (%). Baseline characteristics were balanced by randomization; therefore, no statistical testing was performed on these variables.

SD, standard deviation; BMI, body mass index; ASA, american society of anesthesiologists.

### 5.2 Primary outcome

**FIGURE 2:**
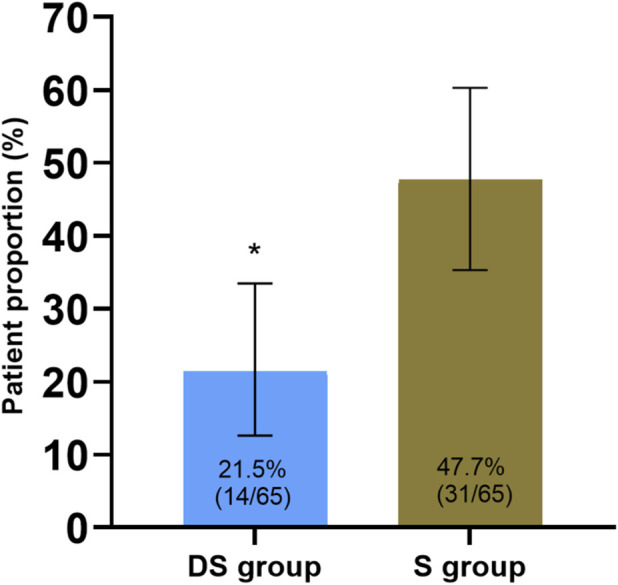
Comparison of the incidence of postoperative sleep disorders (PSQI ≥ 5) on the first postoperative night. Note: The bar chart displays the incidence of sleep disturbance (PSQI global score ≥5) in each group. Values represent percentages (number of occurrences/total number of patients in each group). Error bars indicate the 95% confidence interval. *p < 0.01 compared with the S group.

Analysis of the primary outcome—the incidence of sleep disturbance (defined as a PSQI global score ≥5) on the first postoperative night—revealed that the DS group had a significantly lower incidence rate of 21.5% (14/65; 95% CI: 12.6%–33.5%) compared to 47.7% (31/65; 95% CI: 35.3%–60.3%) in the S group. The absolute risk reduction (ARR) was 26.2%. According to Pearson’s chi-square test, the difference was statistically significant (χ^2^ = 9.822, p = 0.002). The findings of our risk analysis show that the DS group had a lower risk of developing sleep disturbance after surgery compared to that of the S control group (odds ratio, OR = 0.301; 95% CI: 0.140–0.648) (Figure 2).

### 5.3 Secondary outcomes

To compare the PSQI scores, sufentanil consumption and PCA bolus attempts between the two groups in nights 1 and 2 post-operatively, rank-based ANCOVA (covariance analysis) was done. The residual diagnostics revealed that the data had a normal distribution, and the variance homogeneity assumption was violated. Therefore, the Satterthwaite approximation method was employed to compute the degrees of freedom. Significant levels were adjusted using the Bonferroni correction (α = 0.025) for sleep scores involving more than one comparison (both nights). For one comparison (sufentanil consumption and PCA bolus attempts), the significant level was evaluated at α = 0.05.

#### 5.3.1 PSQI scores on postoperative Nights 1 and 2

**FIGURE 3:**
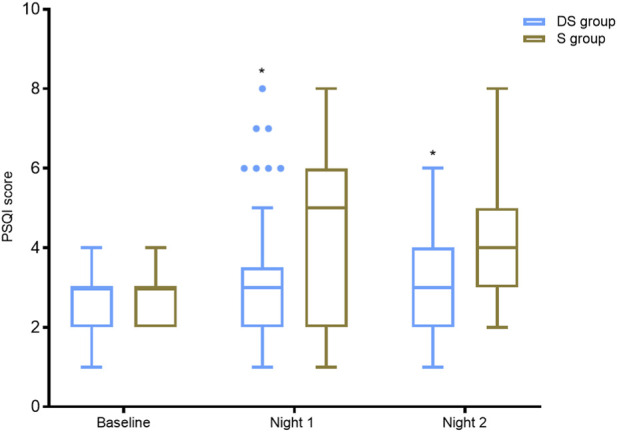
Comparison of Preoperative and Postoperative Sleep Quality (PSQI Scores) Between the Two Groups. Note: Boxplot of Postoperative PSQI Scores for the Two Groups (showing median, interquartile range, whisker range, and outliers). P-values for group comparisons were derived from rank-transformation covariance analysis controlling for surgery type and baseline preoperative scores. *P < 0.025 indicates significant group differences (after Bonferroni correction).

The study’s analysis for PSQI scores revealed a significant main effect for the group. On Night 1 (F (1,125) = 5.548, p = 0.020, partial η^2^ = 0.042) and Night 2 (F (1,125) = 5.947, p = 0.016, partial η^2^ = 0.045), the DS group showed significantly better sleep quality than the S group. Additionally, Surgical type also showed significant main effects: Night 1 (F (1,125) = 5.758, p = 0.018, partial η^2^ = 0.044); Night 2 (F (1,125) = 5.357, p = 0.022, partial η^2^ = 0.041), with laparoscopic patients demonstrating better sleep. Estimated marginal means demonstrated lower scores in the DS group: Night 1 (DS: 54.20, 95% CI [45.65, 62.75] vs. S: 76.96 [68.41, 85.51]); Night 2 (DS: 51.47 [43.23, 59.71] vs. S:79.66 [71.43, 87.90]). Rank-transformed between-group mean differences were significant: Night 1 (−22.76, p = 0.020); Night 2 (−28.19, p = 0.016). No significant group × surgery type interaction was observed (all p > 0.130) (Figure 3).

#### 5.3.2 Sufentanil consumption

**FIGURE 4:**
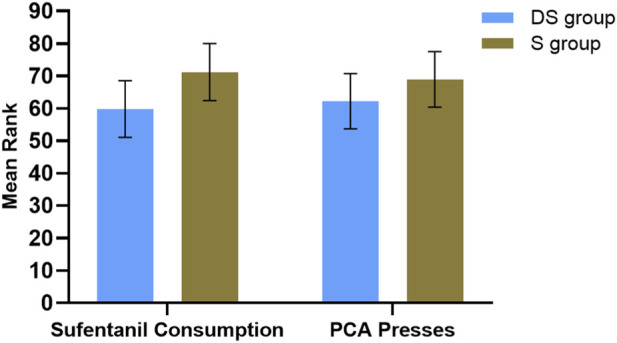
Comparison of Postoperative Sufentanil Consumption and PCA Press Counts. Note: Data are presented as mean ranks from a rank-analysis of covariance, with error bars indicating the standard error of the mean. Higher ranks correspond to greater original values. Between-group comparisons showed no statistically significant differences in sufentanil consumption (p = 0.593) or PCA press counts (p = 0.092).

Analysis of sufentanil consumption showed no significant main effect of group (F (1,126) = 0.288, p = 0.593, partial η^2^ = 0.002). However, a significant main effect of surgery type was found (F (1,126) = 9.065, p = 0.003, partial η^2^ = 0.067), with lower consumption observed in laparoscopic surgery. Estimated marginal means showed no statistically significant difference between groups (DS group: 59.79, 95% CI [51.01, 68.56]; S group: 71.23, 95% CI [62.45, 80.00]; mean difference = −11.44). No significant interaction effect was observed (p = 0.922) (Figure 4).

#### 5.3.3 PCA press counts analysis

Analysis of PCA press counts revealed no significant main effect of group (F (1, 126) = 2.882, p = 0.092, partial η^2^ = 0.022), indicating no statistically significant difference in PCA demand between the DS and S groups, although the effect size suggested a small difference. A significant main effect of surgery type was observed (F (1, 126) = 16.811, p < 0.001, partial η^2^ = 0.118), indicating that surgery type had a significant impact on PCA press counts, with laparoscopic surgery associated with significantly lower counts compared to open surgery. The group × surgery type interaction effect was not significant (F (1, 126) = 2.174, p = 0.143, partial η^2^ = 0.017), suggesting that the group effect was consistent across different surgery types, with a small interaction effect size. Estimated marginal means were: DS group (mean = 62.212, standard error = 4.312, 95% CI [53.679, 70.746]); S group (mean = 68.947, standard error = 4.312, 95% CI [60.413, 77.480]). The between-group mean difference was −6.735; however, the group effect was not statistically significant (p = 0.092), indicating this difference lacked statistical significance (Figure 4).

### 5.4 Linear mixed model analysis of VAS pain scores over 48 hours

**FIGURE 5:**
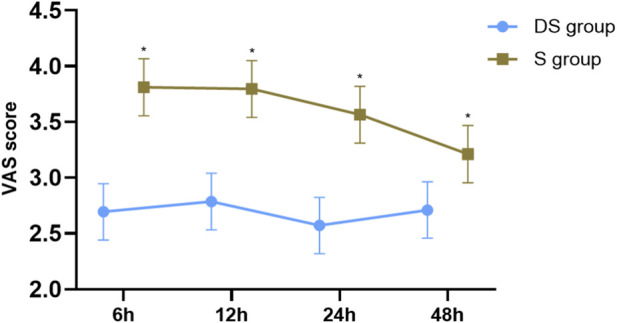
Comparison of VAS scores at different postoperative time points between the two groups. Note: Data are presented as estimated marginal means with 95% confidence intervals, derived from a mixed-effects linear model. *P < 0.025 indicates a significant intergroup difference (Bonferroni-adjusted).

A linear mixed model was used to analyze VAS pain scores during the first 48 h postoperatively. Tests of fixed effects showed significant main effects for group (F (1, 127) = 57.766, p < 0.001), surgery type (F (1, 127) = 18.562, p < 0.001), and time point (F (3, 384) = 4.539, p = 0.004), as well as a significant group × time point interaction (F (3, 384) = 3.523, p = 0.015). Post hoc pairwise comparisons (Bonferroni-adjusted) indicated that the DS group had significantly lower pain scores than the S group at all time points: 6 h (mean difference = −1.117, p = 0.003), 12 h (mean difference = −1.009, p = 0.015), 24 h (mean difference = −0.994, p = 0.018), and 48 h (mean difference = −0.501, p = 0.004). For the main effect of group, the estimated marginal mean pain score was significantly lower in the DS group (2.691, 95% CI [2.512, 2.869]) than in the S group (3.596, 95% CI [3.416, 3.776]), with a mean difference of −0.905 (p < 0.001). For the main effect of time, pain scores changed significantly over time (F (3, 384) = 4.539, p = 0.004). Pairwise comparisons (Bonferroni-adjusted) showed that pain scores at 48 h (estimated marginal mean = 2.960, 95% CI [2.777, 3.144]) were significantly lower than those at 6 h (mean difference = 0.292, 95% CI [0.018, 0.567], p = 0.030) and 12 h (mean difference = 0.331, 95% CI [0.056, 0.605], p = 0.009). The difference between 24 h and 48 h was not statistically significant (mean difference = 0.108, p = 1.000). The significant group × time interaction (F (3, 384) = 3.523, p = 0.015) indicated that the magnitude of the group difference varied over time. Simple effects analysis confirmed that the DS group had significantly lower pain scores than the S group at each individual time point. Specifically, at 6 h, 12 h, 24 h, and 48 h, scores in the DS group were 2.694, 2.787, 2.571, and 2.710, respectively, compared to 3.811, 3.796, 3.565, and 3.211 in the S group. The between-group difference was significantly larger from 6 h to 24 h compared to 48 h, but statistical significance was maintained at all time points. A significant main effect of surgery type was also found (F (1, 127) = 18.562, p < 0.001). Patients undergoing open surgery had significantly higher pain scores (estimated marginal mean = 3.438, 95% CI [3.204, 3.672]) than those undergoing laparoscopic surgery (estimated marginal mean = 2.848, 95% CI [2.712, 2.985]), with a mean difference of 0.590 (p < 0.001) (Figure 5).

### 5.5 Incidence of adverse events

The incidence of adverse events was compared using the Chi-square test or Fisher’s exact test, as appropriate (Table 2).

**TABLE 2:** Comparison of the incidence of postoperative analgesia-related adverse events between the two groups [n (%)].

Outcome	DS group (n = 65)	S group (n = 65)	P-value	Odds ratio (OR)	95% CI
Serious adverse events					
Bradycardia*	11 (16.9%)	2 (3.1%)	0.009	6.42	1.36–30.23
Hypotension	4 (6.2%)	2 (3.1%)	0.680	2.07	0.37–11.69
Other outcomes					
Nausea/vomiting*	9 (13.8%)	21 (32.3%)	0.012	0.34	0.14–0.81
Rescue analgesia*	6 (9.2%)	17 (26.2%)	0.011	0.29	0.11–0.78

Data are presented as n (%). *Significant after hierarchical Bonferroni correction for multiple comparisons (P < .025 within respective tier).

#### 5.5.1 Serious adverse events

- Bradycardia: The bradycardia incidence was higher in DS (11/65, 16.9%) vs. S (2/65, 3.1%) groups (Pearson’s chi-square value = 6.923, p = 0.009). Evidently, none of the bradycardic events fulfilled the pre-specified criteria for pharmacotherapy. All were transient, asymptomatic, and resolved spontaneously or with simple awakening. The risk of bradycardia was significantly greater in the DS group (odds ratio, OR = 6.417, 95% [1.362, 30.228].
- Hypotension: The incidence rate of hypotension was found to be 6.2% in the DS group and 4.6% in the S group. In line with the results for bradycardia, none of the hypotension patients needed a vasopressor according to the pre-defined protocol. The groups did not differ significantly (p = 0.680), and there was no increased risk (OR = 2.066, 95% CI [0.365, 11.692]).

#### 5.5.2 General adverse events

- Postoperative Nausea and Vomiting (PONV): The incidence of PONV was 13.8% (9/65) in the DS group and 32.3% (21/65) in the S group. Intergroup comparison showed a significant difference in the incidence of PONV (Pearson’s chi-square value = 6.192, p = 0.013). Risk analysis indicated that the risk of PONV was significantly lower in the DS group than in the S group (OR = 0.337, 95% CI [0.140, 0.808]).
- Rescue Analgesia (Analgesic Insufficiency): The utilization rate of rescue analgesia was 9.2% (6/65) in the DS group and 26.2% (17/65) in the S group. Intergroup comparison showed a significant difference in the utilization rate of rescue analgesia (Pearson’s chi-square value = 6.392, p = 0.011). Risk analysis indicated that the risk of requiring rescue analgesia was significantly lower in the DS group than in the S group (OR = 0.287, 95% CI [0.105, 0.785]).

## 6 Discussion

In this randomized trial, the addition of low-dose DEX (0.06 μg/kg/h) to a sufentanil-based PCIA regimen improved postoperative sleep quality and pain control, while decreasing the use of rescue analgesia and PONV incidence in gynecological patients. Nonetheless, the aforementioned advantages were observed without a substantive decrease in sufentanil consumption or demand for PCA, indicating a potential mechanism that does not involve opioid sparing. The DEX group had a higher frequency of bradycardia, although these were asymptomatic, reversible and did not lead to clinically significant hypotension. The chosen DEX dose was conservative and corroborated by previous studies demonstrating efficacy and safety in similar dose ranges (Ren et al., 2015; Dong et al., 2016; 2017; Chen et al., 2017).

The enhancement in sleep observed in the DS group was likely due to the multifaceted pharmacological action of DEX against postoperative sleep disturbance. Surgical stress and neuroendocrine-inflammatory response to trauma can significantly alter sleep architecture by suppressing rapid-eye-movement sleep even in the absence of general anaesthesia (Dette et al., 2013). General anesthetics dampen both eye movement (REM) sleep and deep NREM (N3) sleep even further (Butris et al., 2023). DEX functions by activating α2A-adrenoceptors located in the locus coeruleus, leading to inhibition of the ventrolateral preoptic nucleus (VLPO), thereby enhancing the endogenous NREM sleep-promoting pathway and contributing to a more physiological sleep architecture (Nelson et al., 2003). The anxiolytic effect of DEX may have played an add-on role as well, which is particularly relevant for our female subjects. Women are more likely to and do have higher perioperative anxiety, which may be related to estrogen and progesterone levels (Li et al., 2021). The positive correlation of anxiety with postoperative pain and sleep disturbance may have indirectly aided sleep recovery thanks to the mood stabilizer, DEX. This clinical observation is backed by preclinical studies showing a sexually dimorphic anxiolytic response to DEX with more pronounced effects in females (Smith et al., 2013; Jang et al., 2019). On the other hand, the control (S) group’s high incidence of sleep disturbance indicates that opioid-based analgesia has two sides. Opioids interfere with sleep architecture by reducing REM and deep NREM and increasing light sleep and waking. This disruption affects pain tolerance and emotion regulation (Eacret et al., 2020). Clinical studies verify this; for instance, the greater the postoperative opioid consumption, the poorer the sleep quality (Dimsdale et al., 2007; Kjølhede et al., 2012). Therefore, the overall effect of DEX in our regimen appears to be a dual mechanism, where it both directly enhances physiological sleep via CNS mechanisms and simultaneously counteracts the opioid analgesia sleep-inhibiting effect. This action breaks the circle made up of pain, anxiety and poor sleep–a clinically important circle for women in the post-operative phase.

In our gynaecological population, we saw a clear disconnect between improvement in pain score and opioid consumption. The addition of DEX decreased VAS pain scores and consumption of rescue analgesics significantly with no impact on the cumulative intake of sufentanil or PCA demand. This contradiction suggests that the drug’s analgesic effect is not mainly due to opioid-sparing effect. One possible explanation for this dissociation is psychological and sex-related factors. The emotional state of patients influences their perception of pain and their request for analgesics. Preoperative anxiety and depressive symptoms are a better predictor of postoperative pain intensity in women than in men (Franqueiro et al., 2024), and the personality trait of emotional vulnerability helps explain sex differences in the catastrophizing of pain and help seeking (Thorn et al., 2004). This might be reduced by DEX’s mood-stabilizing capabilities. The lower VAS scores and less rescue analgesic usage along with the trend in PCA presses (p = 0.092) indicate that overall subjective analgesic demand was less in DS group despite similar objective opioid consumption. The decoupling of the sensations of pain from the desire to seek out analgesics suggests a modulation of the affective component of pain. Evidence supporting this mechanism comes from studies in postpartum depression showing DEX significantly reduces both the incidence and severity of depressive symptoms in women vulnerable in an emotional sense (Yu et al., 2019). Also, DEX consistently alleviates postoperative emotional dysfunction by acting on the central to regulate mood (Xu S. et al., 2024). This phenomenon can be contextualized further by gender pharmacology. Due to estrogen-mediated modulation of μ-opioid receptor sensitivity (Ji et al., 2007; Lee and Ho, 2013), women often need higher opioid doses than men for similar analgesia. At the same time, sexual dimorphism may influence activation of spinal and supraspinal α2-adrenoceptor by DEX. Studies in animals indicate that estrogen may modulate α2A-adrenergic receptor function which may inhibit anti-nociceptive effect in females (Thompson et al., 2008). A clinical trial studied gender differences and found that a DEX-opioid regimen reduced pain and opioid requirements overall yet men had more of the opioid-sparing benefit. The results also confirmed that women needed considerably higher weight-normalized morphine doses, suggesting that estrogen may inhibit the action of α2-adrenoceptors which mediate analgesia (Li et al., 2016). As a result, the enhanced pain management in DS group can be attributed to DEX’s indirect effects on sleep and mood enhancement which cannot be explained by a direct opioid-sparing effect. Insufficient sleep leads to strong enhancement in pain sensitivity, and sleep disruption after surgery known to result in an increase in pain (Schuh-Hofer et al., 2013). It is likely that DEX breaks this cycle (Wang et al., 2019; Stroemel-Scheder and Lautenbacher, 2023) by inducing restorative sleep, as well as anti-inflammatory effects. This is particularly important in gynecological surgery, where pain is a particular combination of visceral (organ traction) and inflammatory injury (tissue injury) (An et al., 2019). As DEX breaks the cycle of adverse sleep, emotion, and pain, it reduces patients’ pain and their demand for rescue medications mainly via the modulation of the emotional and cognitive-evaluative aspect of pain rather than a direct opioid-sparing mechanism.

With respect to adverse events, the DS group had a better opioid-related safety profile, with significantly lower incidence of PONV compared to S group. Bradycardia occurred almost five times more often in the DS group (16.9% vs. 3.1%), but hypotension rates were similar. The bradycardia rate with DEX is higher, probably due to its sympatholytic action where women seem to be more susceptible because of the higher resting vagal tone associated with estrogen (Abhishekh et al., 2013). The risk of clinically important bradycardia is related to the regimen of administration. Dosing of Loading doses (≥0.5 μg/kg) markedly increase risk (Bharati et al., 2011; Demiri et al., 2019), even the maintenance infusions carry a risk as in a large RCT nearly 19% of patients developed bradycardia, at times severe during laparoscopic pneumoperitoneum (Beloeil et al., 2021), whereas the bradycardia in our study was clinically insignificant. This is probably due to our conservative protocol: a low-dose (0.06 μg/kg/h) postoperative infusion without a loading dose given to younger patients with good cardiovascular reserve. According to recent studies, most of the events were asymptomatic, occurred in sleep, quickly reversed on awakening, and required no intervention which concurs with guideline-recommended symptom-based management (Kusumoto et al., 2019). Hence, bradycardia occurring with this low-dose regimen is a dose-dependent reversible pharmacodynamic effect and not a pathological one. Asymptomatic bradycardia in healthy individuals is not associated with increased cardiovascular risk (Sidhu and Marine, 2020), and its reversibility suggests a vagally-mediated, sleep-like state (Kang et al., 2019). Our even lower dose and stable Ramsay scores (2–3) further support safety. The use of DEX continuous infusion (≤0.08 μg/kg/h) together with sufentanil was not associated with a significantly increased risk of hypotension. Bradycardia risk is higher with DEX; nonetheless, risk remains generally low, and when it occurs, treatment is rarely needed (Feng et al., 2019; Chen et al., 2022). This safety profile justifies a continuous low-dose infusion without a loading dose a technique that has been used with success in other forms of surgery to provide analgesia with improved hemodynamic stability (Zhao et al., 2016; Fan et al., 2017).

The DS group experienced significantly lower rates of postoperative nausea and vomiting (PONV) than the S group (13.8% vs. 32.3%), with similar sufentanil usage. The strong dissociation from opioid-sparing effect supports a direct central antiemetic mechanism of DEX. Preclinical evidence confirms its dose-dependent action via central α2 adrenoceptors (Sun and Darmani, 2024). Importantly, clinical data across doses bolster this independent mechanism: while a higher DEX dose (0.2 g/kg/h) was beneficial as it reduced PONV and opioid demand (Song et al., 2016), an even lower dose (0.01 μg/kg/h) also reduced early PONV without opioid sparing (Li et al., 2020). We chose to employ multimodal antiemetic prophylaxis as per consensus guidelines due to our patients’ high baseline risk (female, gynecologic surgery, postoperative opioids) (Gan et al., 2020). The substantial PONV reduction points to the additive value of including low-dose DEX in such a regimen. The findings of meta-analyses have confirmed DEX can prevent PONV in all types of surgery (Zhang et al., 2022). Continuous infusion, with no loading dose, is also sufficiently antiemetic without causing cardiovascular complications (Jin et al., 2017). Also, as gynecological patients suffer from other aggravating factors, including the steroidal hormonal effects throughout the menstrual cycle, preoperative anxiety and genetic polymorphisms, we require all of these strategies (Echeverria-Villalobos et al., 2022).

## 7 Conclusion

To sum up, the addition of a low-dose of dexmedetomidine (0.06 μg/kg/h) to a sufentanil-based PCIA regime improves postoperative sleep quality and reduces pain scores, need for rescue analgesics and antiemetics in patients undergoing gynecological surgery without any opioid-sparing effect. The advantages are thought to arise mainly from DEX’s ability to level moods and encourage sleep, potentially reducing the vicious cycle of pain-sleep disturbance. The regimen was associated with a greater frequency of asymptomatic clinically tolerable bradycardia but without increased risk of hypotension. Consequently, low-dose DEX is an important and effective non-opioid-sparing component of multimodal analgesia after gynecological surgery. Future studies should establish optimal dosing for specific subpopulations and assess functional outcomes at longer-term.

## 8 Limitations

Our data were obtained from a single-centre cohort of 130 otherwise healthy women, and may not be generalizable to older, multi-morbid, or ASA III-IV patients. The patients’ PSQI reports were solely used to assess sleep. The PSQI has been used in postoperative sleep studies evaluating the first and second nights (Wang et al., 2023; Yan et al., 2023). However, the PSQI is not as sensitive as daily NRS to capture slight variations from day to day. As there is no polysomnography, we cannot rule out recall or expectation bias nor relate slight sleep-stage changes to synchronous anxiety variations. Moreover, we did not assess anxiety quantitatively before or after surgery, which limits direct examination of the proposed anxiolytic pathway as a mechanism for DEX’s benefits. Since we did not document the menstrual-cycle phase, contraceptive use, or perioperative sex-hormone levels, any hormone-mediated modulation of DEX’s effects remains theoretical and requires direct confirmation. The DS group had a higher incidence of bradycardia. However, intermittent ward ECGs may have missed transient asymptomatic arrhythmias. Therefore, continuous ambulatory ECG monitoring in a larger, more diverse cohort is needed before deeming this regimen safe for patients with potential cardiac disease.

## Data Availability

The raw data supporting the conclusions of this article will be made available by the authors, without undue reservation.
